# A problem-solving task specialized for functional neuroimaging: validation of the Scarborough adaptation of the Tower of London (S-TOL) using near-infrared spectroscopy

**DOI:** 10.3389/fnhum.2014.00185

**Published:** 2014-03-28

**Authors:** Anthony C. Ruocco, Achala H. Rodrigo, Jaeger Lam, Stefano I. Di Domenico, Bryanna Graves, Hasan Ayaz

**Affiliations:** ^1^Clinical Neurosciences Laboratory, Department of Psychology, University of Toronto ScarboroughToronto, ON, Canada; ^2^School of Biomedical Engineering, Science and Health Systems, Drexel UniversityPhiladelphia, PA, USA

**Keywords:** functional near-infrared spectroscopy (fNIRS), Tower of London, validation, dorsolateral prefrontal cortex, problem-solving, executive functioning, deliberation

## Abstract

Problem-solving is an executive function subserved by a network of neural structures of which the dorsolateral prefrontal cortex (DLPFC) is central. Whereas several studies have evaluated the role of the DLPFC in problem-solving, few standardized tasks have been developed specifically for use with functional neuroimaging. The current study adapted a measure with established validity for the assessment of problem-solving abilities to design a test more suitable for functional neuroimaging protocols. The Scarborough adaptation of the Tower of London (S-TOL) was administered to 38 healthy adults while hemodynamic oxygenation of the PFC was measured using 16-channel continuous-wave functional near-infrared spectroscopy (fNIRS). Compared to a baseline condition, problems that required two or three steps to achieve a goal configuration were associated with higher activation in the left DLPFC and deactivation in the medial PFC. Individuals scoring higher in trait deliberation showed consistently higher activation in the left DLPFC regardless of task difficulty, whereas individuals lower in this trait displayed less activation when solving simple problems. Based on these results, the S-TOL may serve as a standardized task to evaluate problem-solving abilities in functional neuroimaging studies.

## Introduction

**Figure 1:**
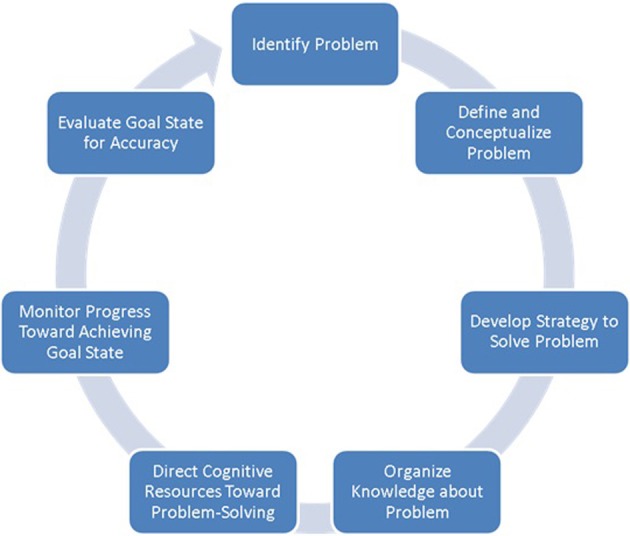
**Stages typically considered as part of the problem-solving cycle (adapted from Pretz et al., 2003)**.

The term *problem-solving* refers to a multifaceted higher-order cognitive function directed toward identifying problems with the current state, and generating and implementing potential solutions to achieve a goal state (Simon and Newell, 1971). Problem-solving is considered a cyclical process comprising several interacting non-sequential stages (Figure 1). Broadly, problem-solving can be summarized to involve three partially overlapping cognitive operations: problem recognition, definition, and representation (Pretz et al., 2003). Problem *recognition* concerns the extent to which a problem is directly presented to an individual, or if it requires discovery or creation by the problem-solver (Getzels, 1982). Problem *definition* refers to the precision with which a problem's scope and goals are delineated, ranging from a problem that is clearly defined to one that is indistinct, the former usually associated with a presented problem and the latter with one that is discovered or created by the problem-solver. Last, problem *representation* is thought to encompass four components: a description of the initial state of the problem, a description of the goal state, a set of allowable operators (i.e., actions taken to move from one state to another), and a set of constraints (Pretz et al., 2003).

Several cognitive tests have been developed to assess problem-solving ability in an objective and standardized manner. *Tower tests* are among the most commonly administered tests of problem-solving for both research and clinical purposes (for a review, see Sullivan et al., 2009). These tasks normally present individuals with three placeholders (usually pegs or pockets) and multiple balls or discs which can be put onto each placeholder. Conventional tower tasks typically differ in the lengths of the placeholders (equal or unequal lengths), colors of the balls or discs (monochromatic or polychromatic), and sizes of the balls or discs (equal or unequal circumferences). Based on the three-component model of problem-solving described by Pretz et al. (2003), the conventional tower task can be characterized as a presented problem (*recognition*) whose initial and goal states, allowable operators, and constraints, are precisely demarcated (*definition*). Features which typically differ from one variant of the task to another, however, are the number of allowable operators required to achieve the goal state, and the specific constraints (or “rules”) imposed on the problem-solving task (*representation*). Therefore, conventional tower tasks do not measure the cognitive processes underlying discovered or created problems (i.e., problem types that are more challenging to evaluate in a controlled and standardized fashion). Perhaps due to their narrow problem definition and ease of standardization, tower tasks have enjoyed widespread clinical application (Culbertson and Zillmer, 1998; Culbertson et al., 2004) and extensive validation to identify the component cognitive functions which may underlie performance on these tasks (Welsh et al., 1999; Unterrainer et al., 2004).

Researchers attempting to understand the neural underpinnings of problem-solving ability have adapted a variety of paper-and-pencil tower tasks for administration by computer. Perhaps the most commonly adapted tower task is known as the Tower of London (TOL) (Shallice, 1982). Modifications to this task have typically involved changes to its representational features, namely, the number of allowable operators to achieve the goal state and the mode by which allowable operators may be implemented (i.e., not by physically moving the balls or discs from one placeholder to the next, but instead by mentally visualizing each move). Using functional magnetic resonance imaging, functional near-infrared spectroscopy (fNIRS) and positron emission tomography, several studies have used these tasks to delineate the neural circuitry underlying problem-solving ability^1^ (Table 1). While the parameters for each variant of the TOL varied from one study to another, the prefrontal cortex (PFC) was reliably activated across all studies, including its anterior, inferior, and dorsolateral (DLPFC) aspects (Baker et al., 1996; Boghi et al., 2006; Wagner et al., 2006; Just et al., 2007; Den Braber et al., 2008; Fitzgerald et al., 2008; Campbell et al., 2009; Zhu et al., 2010; De Ruiter et al., 2011; Kaller et al., 2011; Stokes et al., 2011; Hahn et al., 2012). Activation was also routinely observed in a number of other cortical and subcortical regions, including the parietal cortex, premotor region, anterior cingulate cortex, insular cortex, caudate, and thalamus (Baker et al., 1996; Beauchamp et al., 2003; Cazalis et al., 2006; Just et al., 2007; Campbell et al., 2009; Den Braber et al., 2010). With increasing “difficulty” or “task load” (i.e., the greater the number of allowable operators, or “moves,” required to achieve the target configuration), higher levels of activation were observed primarily within the left DLPFC as well as the parietal cortex bilaterally (Rasmussen et al., 2006; Den Braber et al., 2008, 2010), areas which also have showed significant functional connectivity during performance on this task (Just et al., 2007). The left DLPFC has been linked specifically to the extraction of goal information and the generation of an internal problem representation, whereas the right DLPFC may be more strongly associated with working memory and mental transformations (Newman et al., 2003, 2009; Van Den Heuvel et al., 2003; Wagner et al., 2006; Ruh et al., 2012).

**Table 1:** **Task parameters for computerized adapted versions of the Tower of London used in functional neuroimaging studies published since 2000**.

**Study**	**Imaging technique**	**Placeholders**	**Minimum moves on target trials**	**Baseline or control task**	**Accuracy range (% correct)**	**Response format**
Den Braber et al., 2008	fMRI	Unequal pegs	1–5	Count number of balls on the display	~100% (1-move problems) to ~70% (5-move problems)	Two-alternative forced-choice manual response button selection
Campbell et al., 2009	fMRI	Unequal pegs	Did not specify	Passive viewing of display	100%	Three response buttons (one for each peg), with a first press selecting the peg and its topmost ball, and a second press indicating the location where ball is to be placed
Cazalis et al., 2003	fMRI	Unequal pockets	2–6	0- and 1-move problems	~99% (0- and 1-move problems) to ~67% (4-, 5-, and 6-move problems)	Manual response button selection of 7 response alternatives
Dagher et al., 1999	PET	Unequal pockets	1–5	Blank computer screen	~100% (1-move problems) to 46% (5-move problems)	Manual on-screen selection of ball and then location where ball is to be placed
Fitzgerald et al., 2008	fMRI	Pegs (sizes not specified)	Did not specify	Fixation crosshair	Did not specify	Two-alternative forced-choice manual response button selection
Hahn et al., 2012	fMRI and PET	Unequal pegs	2–8	Fixation crosshair	Did not specify	Two-alternative forced-choice manual response button selection
Just et al., 2007	fMRI	Unequal pockets	2–3	1-move (70%) and 2-move (30%) problems	92% (2- and 3-move problems)^a^	Manual response button selection of 4 response alternatives
Kaller et al., 2011	fMRI	Equal pegs	3	Did not specify	~97% (3-move problems)	Manual response button selection of ball and then location where ball is to be placed
Newman et al., 2003	fMRI	Unequal bins	1–6	Fixation crosshair	90% (did not specify accuracy by number of moves required to solve problem)	Manual response button selection of 4 response alternatives
Rasmussen et al., 2006	fMRI	Pegs	3–5	Scrambled image	88% (did not specify accuracy by number of moves required to solve problem)	Manual response button selection of 3 response alternatives
Ruh et al., 2012	fMRI	Pegs	3	Did not specify	Did not specify	Manual response button selection of 3 response alternatives
De Ruiter et al., 2009b	fMRI	Pegs	1–5	Count number of balls on the display	Did not specify	Two-alternative forced-choice manual response button selection
Stokes et al., 2011	fMRI	Unequal pockets	Did not specify	Count number of balls on the display	Did not specify	Did not specify
Wagner et al., 2006	fMRI	Unequal pegs	2–5	Count number of balls on the display	95% (2-move problems) to 82% (5-move problems)	Manual response button selection of 4 response alternatives
Zhu et al., 2010	fNIRS	Unequal pegs	1–4	0-move problems	Insufficient information to calculate	Verbal response

*fMRI, functional magnetic resonance imaging; fNIRS, functional near-infrared spectroscopy; PET, positron emission tomography*.

a *Data reported are for healthy control participants*.

Taken together, these studies have provided important information about the role of the DLPFC in problem-solving on the TOL. These investigations, however, incorporated adapted computerized variants of this task that varied along a number of task parameters (see Table 1) that could influence the patterns of DLPFC activation observed in one study to another. These included the number of “moves” (or allowable operators) required to achieve the target configuration (which varied from 2 to 6), the mode of responding (which ranged from a forced-choice two-alternative format to one which required examinees to press one of seven buttons on a keypad), and the baseline or “control” task used for comparison with more complex problem-solving trials (which ranged from a blank screen or fixation crosshair to the use of 0- and 1-move problems). Variations in these task parameters could contribute to discrepant findings observed across studies, potentially obscuring subtle distinctions in DLPFC activation patterns that may be associated with dissociable cognitive functions.

The primary aims of the present study were to develop and validate a new computerized version of the TOL designed specifically for neuroimaging, called the Scarborough adaptation of the Tower of London (or S-TOL). An experimental task like the S-TOL is said to be valid for measuring an intended cognitive function when it produces measurement outcomes (e.g., patterns of neural activation) that are consistent with the theory of response behavior that guided its construction (Borsboom et al., 2004; Borsboom, 2005). Given that the S-TOL was developed to measure problem-solving ability using an established paradigm associated with a reasonably well-defined pattern of regional brain activation, we anticipated that similar neural activations would be elicited by the S-TOL to provide converging support for its validity as a problem-solving task.

Validity of the S-TOL was also examined on the basis of the relationship between a trait known as deliberation and activity in the DLPFC across levels of task difficulty (i.e., lower vs. higher). *Deliberation* is defined as “the tendency to think carefully before acting” (McCrae and Costa, 2010, p. 24). Within the context of a laboratory task like the S-TOL, we expected participants who are disposed to engage with problem-solving items in a more thoughtful manner (i.e., individuals higher in deliberation) to exhibit a more consistent pattern of activation within those PFC regions that are critical for problem-solving (i.e., DLPFC), regardless of task difficulty. This association between deliberation and task difficulty would constitute further evidence that the S-TOL is valid for studying problem-solving processes by demonstrating that response behavior on the S-TOL varies systematically with the quality of task engagement to which respondents are disposed on problem-solving tasks.

Importantly, the S-TOL was intentionally designed with an eye toward its potential for translation to clinical samples (i.e., individuals with psychiatric and neurological disorders). Therefore, the task was constructed with the aim of achieving high levels of accuracy, even in individuals with possible central nervous system dysfunction, to reduce the likelihood of frustration on the task and to facilitate comparisons between activation blocks containing lower and higher difficulty problems. This approach in designing the S-TOL was considered essential to ensure that any observed differences in patterns of brain activation between clinical and non-clinical groups are not confounded with differences in accuracy on the task. Accordingly, this task incorporated problems requiring no more than three moves to achieve a target configuration, and utilized a simplified response format (i.e., a forced-choice “yes” or “no” answer to the same task instruction across all trials), thereby reducing the need for complex response devices and minimizing demands on working memory. The development and validation of a standardized tower task that can be readily incorporated into neuroimaging protocols could increase consistency of methods across studies and facilitate comparisons of results between clinical and non-clinical samples.

## Materials and methods

### Participants

An initial sample of 43 healthy adults provided informed written consent to participate in this study. Four participants were subsequently excluded because they obtained atypically inaccurate performances (<60%) on zero-move (ZM) trials of the S-TOL (i.e., these participants may not have understood or complied with instructions), and one participant was excluded due to technical problems with an event-marker file for the neuroimaging task. The final sample comprised 38 adults who were recruited from the University of Toronto Scarborough's undergraduate research participant pool as well as the surrounding University community. Participants were largely right-handed (76.3%) and female (60.5%) with an average 13.8 years (*SD* = 1.7) of formal education. The ethno-racial composition of the sample according to 2011 Canadian census categories was as follows: Chinese (34.2%), White (21.1%), South Asian (13.2%), Black (5.3%), Filipino (5.3%), Latin American (5.3%), Japanese (2.6%), Korean (2.6%), Southeast Asian (2.6%), West Asian (2.6%), and Other (5.3%). Prior to commencing the neuroimaging protocol, participants completed a brief screening measure to collect demographic information and to rule out the presence of any serious manual, ophthalmic, neurologic (i.e., seizure disorder, severe head injury), or psychiatric illness (i.e., psychosis, bipolar disorder).

### Procedure

**Figure 2:**
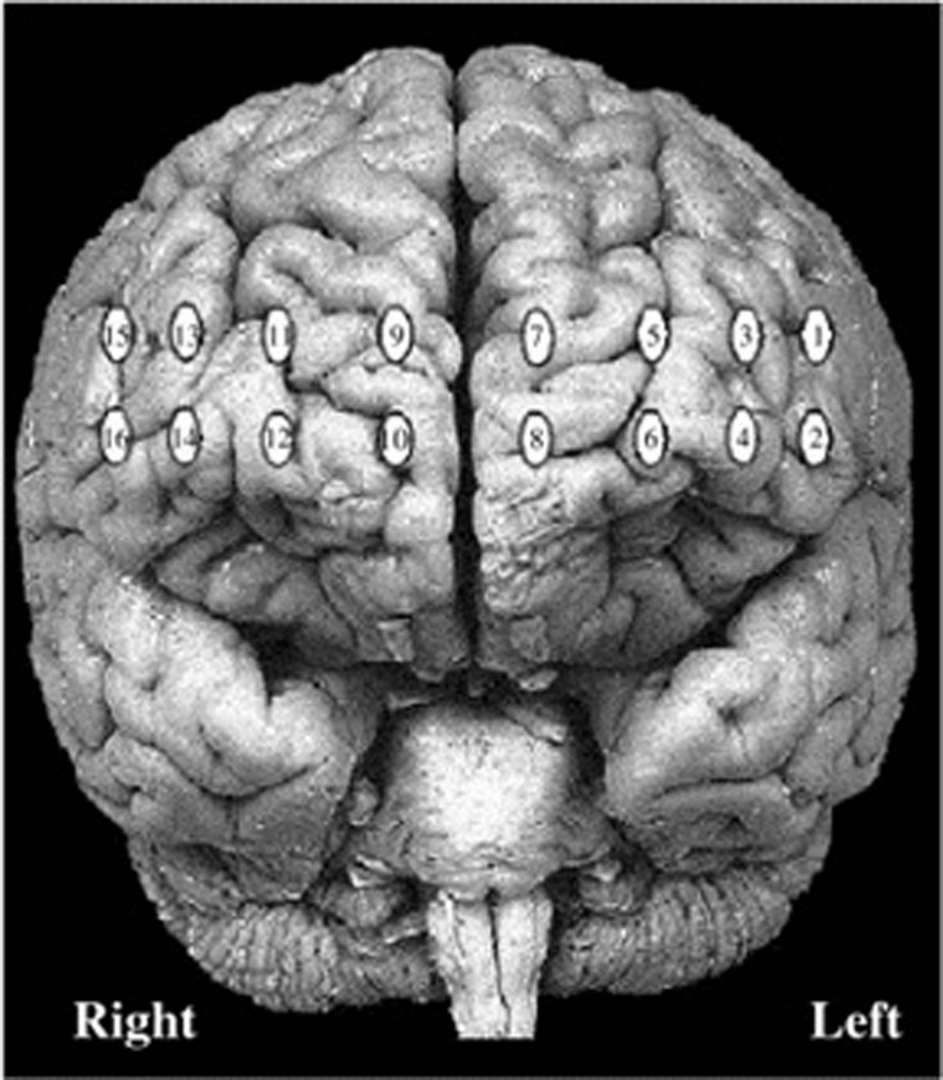
**Locations of 16 channels for continuous-wave functional near-infrared spectroscopy system**.

This research was conducted in accordance with Canada's 2nd edition of the Tri-Council Policy Statement: Ethical Conduct for Research Involving Humans and was approved by the Social Sciences, Humanities and Education Research Ethics Board at the University of Toronto. After a complete description of the study, participants were seated in a dimly-lit room in front of a computer monitor and a keyboard. Participants were asked to sit comfortably and interact with the computer using the mouse with their right hand. Prior to beginning each task, instructions were presented on the monitor and read aloud by the experimenter. Testing did not proceed until participants acknowledged that they understood all instructions completely. After finishing all procedures, participants were compensated for their time with course credit or $10 for each hour of the experiment. After the participant's forehead was cleaned using an alcohol swab, the fNIRS probe was positioned over the forehead and secured at the back of the head using Velcro® straps. The fNIR Imager 1000® (fNIR Devices, Potomac, MD) is a continuous-wave fNIRS system described in previous studies conducted by our research group (Ruocco et al., 2010; Ayaz et al., 2012; Rodrigo et al., 2014). Two wavelengths of light (730 and 850 nm) were measured continuously at 500 ms intervals in 16 channels with 1.25 cm penetration. The probe was aligned with the electrode positions F7, FP1, FP2, and F8 (which correspond to Brodmann areas 9, 10, 45, and 46) based on the international 10–20 EEG system (Jasper, 1958). Specific details regarding probe placement are provided in Ayaz et al. (2006). Figure 2 displays the spatial location of each channel of the fNIRS system. Image reconstruction was rendered using the topographic tools available in fNIRSoft® Professional Edition (Ayaz, 2010), which provides spatial visualization of fNIRS activation data using magnetic resonance imaging templates as described in Ayaz et al. (2006). After completing fNIRS procedures, participants were seated in a testing room and asked to complete a personality inventory that evaluated traits related to decision-making and impulsiveness.

### Scarborough adaptation of the tower of london (S-TOL)

**Figure 3:**
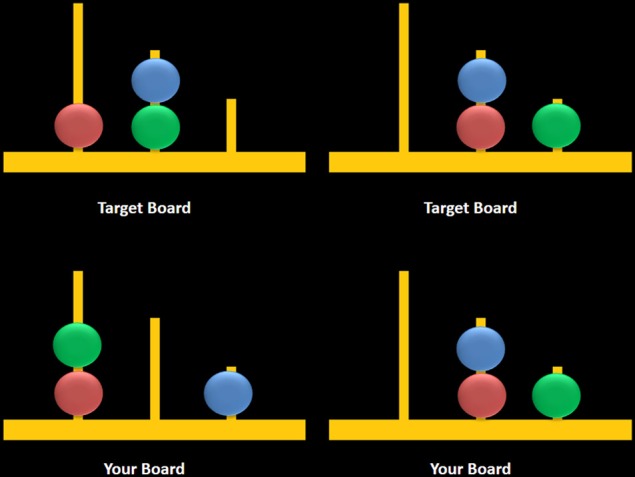
**Sample computerized stimuli from the Scarborough adaptation of the Tower of London task**. The left panel displays sample problems requiring a minimum of two moves to solve the problem (left) or zero moves to solve the problem (right). Stimuli were designed based on descriptions provided in Shallice (1982).

Participants completed a newly adapted computerized version of the TOL which was designed according to original descriptions of this task as presented in Shallice (1982). Two boards were visually presented in color on a computer screen (Figure 3). The task began with the following on-screen instructions which were also read aloud by the examiner:

On this task, you will see two boards: one at the top of the screen and one at the bottom. The board at the top of the screen is called the target bard and the board at the bottom of the screen is your board. Each peg has a different size. The first peg can hold three colored balls. The second peg can hold two colored balls. The third peg can hold one colored ball. Your job is to decide how many times you need to move the colored balls, from one peg to another, to make your board look like the target board. You will have 7 s to study the two boards, afterward; you will always be asked the same question: Can you solve this in exactly two moves? You will have 3 s to decide your answer.

Participants were also provided with two rules: (1) they could move only one ball at a time, and (2) they could not put more balls on a peg than what it could hold. Participants completed three practice trials and had the opportunity to ask questions about the task. After the practice trials, participants were given the following instruction:

Remember to stay as still as you can, and to keep your hand on the mouse at all times. Think through each problem carefully. Be sure to decide yes or no as accurately as possible within the 3-s time limit. It is more important to be accurate in your decision than to give your answer quickly.

The task consisted of two trial types: multiple-move (MM) trials, which included problems that could be completed in a minimum of either two or three moves, and ZM trials, during which participants observed two boards displaying identical configurations (i.e., no moves were required) (Figure 3, right panel). MM trials included a combination of problems that either did or did not require an intermediate step to achieve the goal state (Figure 3, left panel). The correct answer was “yes” for two-move trials and “no” for three-move and ZM trials, the latter included as a check to ensure that participants remained engaged during ZM trials. The advantage of using the same instruction for MM and ZM trials was that the maintenance of this instruction in working memory was equivalent between trials. Each trial began with a 7-s study period which was followed by a 3-s window during which participants were asked to respond using a mouse by clicking on a box labeled either “yes” or “no.” Trials were grouped into blocks containing six of either MM or ZM trials, and blocks were separated by a 30-s rest period when participants were asked to fixate on a crosshair at the center of the screen. Blocks were alternated, starting with a MM block, over a total of six repetitions for each block. Accuracy and response times (RT) were recorded for MM and ZM trials.

### Trait deliberation

Participants completed the NEO Personality Inventory-3 (NEO-PI-3; McCrae et al., 2005a), a 240-item self-report personality inventory that was designed to measure the major domains of personality based on the Five-Factor Model (FFM) of personality (McCrae and Costa, 1987). Of interest to the current study was a trait facet scale, referred to as *Deliberation*, which reflects the tendency for individuals to think things through before acting or speaking. Examinees are asked to rate their answers to items on a five-point Likert-type scale: strongly disagree, disagree, neutral, agree, and strongly agree. The NEO-PI-3 shows strong convergence and similar (if not superior) psychometric properties when compared to its predecessor, the NEO-PI-Revised (McCrae et al., 2005b; De Fruyt et al., 2009). These measures have demonstrated excellent reliability and validity across a large number of studies (see McCrae et al., 2005a).

### Statistical analyses

#### Signal processing

Raw fNIRS light intensities were manually screened to exclude channels which had poor signal quality (i.e., very low signal or saturation) and subsequently underwent signal processing to exclude physiological artifacts using a low-pass filter with a finite impulse response and a linear phase filter with an order of 20 and a cut-off frequency of 0.1 Hz (Izzetoglu et al., 2004; Ayaz et al., 2012). Following these procedures, channels that were identified as being problematic using a sliding-window motion artifact rejection (SMAR) technique (Ayaz et al., 2010) were analyzed and confirmed rejected through visual inspection. On average, 2.67 (*SD* = 2.06) channels were excluded for each participant primarily because of saturation with ambient light when contact with the skin was not optimal or in lateral channels due to interference from hair shafts. Time synchronization markers denoting the beginning and end of each block were delivered to the fNIRS acquisition device using a serial connection. Based on the markers that separated MM and ZM blocks, data for local baseline segments and activation segments were extracted and compared. The activation segments consisted of blocks (i.e., ZM and MM) that were 60-s in duration. Each block consisted of 6 trials that began with a 7-s observation period and a 3-s response period. The local baseline segments included the first 10-s of each block, representing 20 observations sampled at 500-ms intervals at the beginning of each block. The primary measure of interest in this study was oxygenated hemoglobin (oxy-Hb), although deoxygenated hemoglobin and total hemoglobin measurements were also collected but not reported because oxy-Hb is more commonly associated with neural activity.

#### Statistical plan

In order to control for neural activation associated with visual attention and working memory (i.e., maintaining task instructions in mind), primary analyses contrasted ZM and MM blocks. Therefore, the main difference between ZM and MM blocks was that the latter required participants to solve problems that required a minimum of two or three moves (rather than none) to achieve the goal state. According to the Related-Samples Wilcoxon Signed Rank Test, participants made more errors on MM as compared to ZM trials, *z* = 4.19, *p* < 0.001, *r* = 0.70. To control for differences in accuracy between MM and ZM blocks, a criterion of 90% correct responses was applied to both trial types. This procedure identified a subset of participants (*n* = 24) that obtained similar performances across MM and ZM conditions, *t*_(23)_ = 2.01, *p* = 0.05. Contrasts of activation associated with MM and ZM conditions for these highly accurate participants (*n* = 24) were visualized separately from the less accurate participants (*n* = 14) to highlight differences in functional activation between these groups. All data were visualized on a standard MRI template (Figure 2).

The fNIRS time-series data were analyzed with multilevel models (Bryk and Raudenbush, 1992; Kenny et al., 1998). Multilevel models are regression models that feature fixed effects as well as random effects (i.e., parameters distributed according to some probability distribution). Multilevel models confer a number of advantages over traditional repeated measures ANOVA. Two such advantages that are relevant to the present investigation concern the inclusion of unbalanced data and the flexibility to incorporate continuous predictors. Multilevel models have been recommended for psychophysiological research (Bagiella et al., 2000), and have been employed in our previous studies using fNIRS (e.g., Di Domenico et al., 2013; Rodrigo et al., 2014).

Within the context of the present study, multilevel models take into account that the data points comprising each participant's experimental time-series measurements (i.e., oxy-Hb measurements taken at intervals of 500-ms) are nested within the respective participants and that the number of data points may be unbalanced across participants due to signal processing. Thus, variance in the dependent variable (i.e., oxy-Hb) is partitioned into within-person (Level-1) and between-person (Level-2) components, allowing predictor terms to be represented at both the level of the experimental condition (i.e., the MM and ZM conditions) and at the level of the participant, respectively. In the primary analyses, we examined the Level 1 effect of problem-solving difficulty on the S-TOL across 16 fNIRS channels, controlling for Type I error (*p* < 0.05) using the False Discovery Rate (FDR) approach (Benjamini and Hochberg, 1995). Problem-solving was effect-coded (*ZM* = −1; *MM* = 1) in all multilevel analyses. Thus, a two-unit change on this effect-code represents the unstandardized mean difference in oxy-Hb across the ZM and MM conditions.

All multilevel models were estimated in R Core Team (2013) using the *multilevel* and *nlme* packages (Bliese, 2009). We estimated random intercept models, nesting the experimentally demarcated time-series data within each participant. To account for the temporal autocorrelation in the time-series, all models were conservatively estimated using an unstructured covariance matrix and the “between-within” method of estimating degrees of freedom (Schluchter and Elashoff, 1990). The descriptive statistics for the oxy-Hb time-series are provided in Table 2. The intraclass correlations across the fNIRS channels ranged from 0.03 to 0.28 indicating a small but significant degree of dependence among participants' nested data points and a substantial amount of within-person variation during the time-course of the S-TOL as expected.

**Table 2:** **Descriptive statistics for oxy-Hb time-series across fNIRS channels**.

**fNIRS Channel**	**Intraclass correlation**	***N***	***n***
1	0.08	28778	27
2	0.11	34504	33
3	0.07	38804	34
4	0.05	33060	33
5	0.09	40922	38
6	0.06	35288	34
7	0.08	38783	37
8	0.28	34195	34
9	0.06	38737	37
10	0.13	33228	34
11	0.04	31376	32
12	0.06	37949	37
13	0.03	33877	31
14	0.04	38993	37
15	0.05	24931	23
16	0.15	41220	38

*All intraclass correlations are significant at p < 0.0001. N = total number of data points, aggregated across all participants; n = total number of participants with available data. The numbers of data points across fNIRS channels are unbalanced due to filtering*.

## Results

### Behavioral performance and personality traits

On the S-TOL, all participants (*N* = 38) attained 97.5% (*SD* = 3.72) accuracy on ZM problems, 88.9% (*SD* = 15.8) on two-move problems, and 89.2% (*SD* = 12.73) on three-move problems. Mean RT was 161 ms (*SD* = 914) for correct ZM problems, 896 ms (*SD* = 155) for two-move problems, and 1018 ms (*SD* = 206) for three-move problems.

*T*-scores (with a normative mean of 50 and *SD* of 10) for the participants' self-reported levels of deliberation as measured by the NEO-PI-3 was within normal limits (*M* = 45.1, *SD* = 10.7). There were no significant correlations (Spearman's rho) between Deliberation scores and accuracy indices from the S-TOL (Table 3; all *p*'s > 0.05).

**Table 3:** **Spearman's rho correlations between impulsive personality traits and accuracy on the Scarborough adaptation of the Tower of London**.

	**Deliberation**	**0-Move accuracy**	**2-Move accuracy**
0-Move accuracy	0.13		
2-Move accuracy	0.11	−0.01	
3-Move accuracy	0.07	0.15	0.29

### Solving complex vs. simple problems

**Figure 4:**
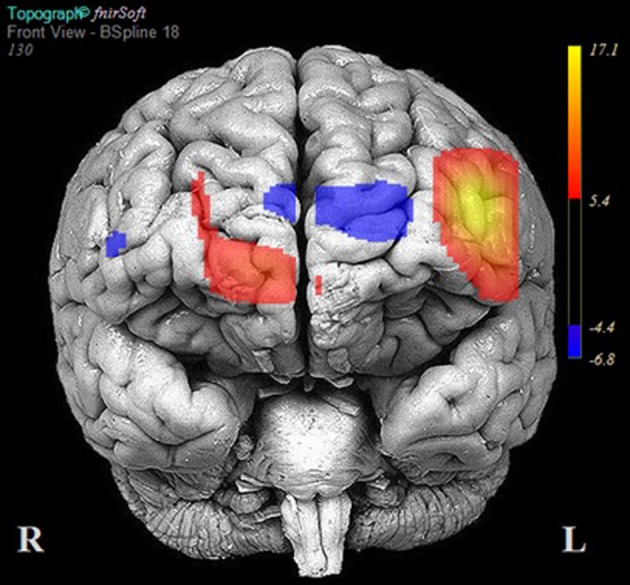
**Areas of significant activation and deactivation associated with solving two- and three-move problems on the Scarborough adaptation of the Tower of London (S-TOL) task for all participants (*N* = 38)**. Areas of significant activation are denoted in red, and areas showing significant deactivation are in blue. Data represent *t*-scores for the contrast of multiple-move and zero-move conditions (*p* < 0.05, False-Discovery Rate-corrected).

The results of multilevel analyses comparing oxy-Hb across the MM and ZM conditions for all participants (*n* = 38) is presented in Table 4. Significantly greater increases in oxy-Hb for MM compared to ZM were observed in two distinct clusters, the first encompassing the anterior aspects of the left inferior/middle frontal gyrus (left DLPFC; channels: 1, 2, 3, and 4), and the second, the right superior frontal gyrus (right medial PFC channels: 10, 11, and 12). Conversely, significantly less oxy-Hb for MM as compared to ZM conditions was observed in two clusters, the first centered over the medial PFC (channels 5, 7, and 9), and the second comprising a single channel (16) over the most anterior aspect of the right inferior frontal gyrus (IFG). Channels 6, 8, 13, 14, and 15 showed no difference in oxy-Hb changes across the MM and ZM conditions. These results are displayed in Figure 4.

**Table 4:** **Multilevel analyses comparing oxy-Hb levels for multiple-move and zero-move conditions for all participants (*N* = 38)**.

**fNIRS Channel**	***b***	***SE***	***df***	***T***
1	0.4163	0.0036	28750	11.67^**^
2	0.0392	0.0039	34470	9.96^**^
3	0.0458	0.0027	38769	17.14^**^
4	0.0265	0.0035	33026	7.50^**^
5	−0.0165	0.0026	40883	−6.35^**^
6	0.0003	0.0035	35253	0.11
7	−0.0208	0.0030	38745	−6.83^**^
8	0.0014	0.0039	34160	0.36
9	−0.0160	0.0027	38699	−5.99^**^
10	0.0231	0.0033	33193	7.01^**^
11	0.0178	0.0033	31343	5.42^**^
12	0.0169	0.0030	37911	5.59^**^
13	0.0009	0.0028	33845	0.34
14	0.0061	0.0029	38955	2.07^*^
15	0.0028	0.0036	24907	0.77
16	−0.0156	0.0035	41181	−4.45^**^

** p < 0.001,

* *p < 0.05. All models were estimated with an unstructured covariance matrix and the between-within method of estimating degrees of freedom. Significance levels are FDR corrected*.

### Highly accurate vs. less accurate problem-solving

**Figure 5:**
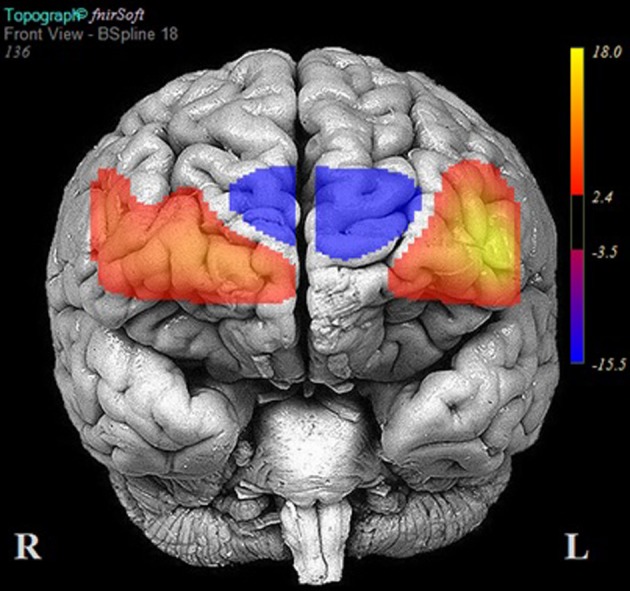
**Areas of significant activation and deactivation associated with solving two- and three-move problems on the Scarborough adaptation of the Tower of London (S-TOL) task for participants matched for high accuracy (*N*= 24)**. Areas of significant activation are denoted in red, and areas showing significant deactivation are in blue. Data represent *t*-scores for the contrast of multiple-move and zero-move conditions (*p* < 0.05, False-Discovery Rate-corrected).

Table 5 reports the results of multilevel analyses comparing oxy-Hb across the MM and ZM conditions for participants who were matched for accuracy at the 90% accuracy threshold (*n* = 24). Significant increases in oxy-Hb compared to ZM were observed in 12 channels, encompassing the anterior aspects of the right (channels: 10, 11, 12, 13, 14, 15, and 16) and left (channels 1, 2, 3, 4, and 6) DLPFC. Conversely, significant decreases in oxy-Hb compared to ZM were observed in four channels encompassing the medial PFC (channels: 5, 7, 8, and 9). The results of these analyses are portrayed in Figure 5.

**Table 5:** **Multilevel analyses comparing oxy-Hb levels for participants who were matched for high accuracy (*N* = 24)**.

**fNIRS Channel**	***b***	***SE***	***df***	***t***
1	0.0243	0.0039	19869	6.23^**^
2	0.0769	0.0043	23884	17.97^**^
3	0.0342	0.0029	24133	11.60^**^
4	0.0518	0.0041	20621	12.71^**^
5	−0.0256	0.0031	26675	−8.29^**^
6	0.0156	0.0038	24758	4.13^**^
7	−0.0574	0.0037	25351	−15.46^**^
8	−0.0172	0.0048	21913	−3.57^**^
9	−0.0424	0.0028	25329	−15.05^**^
10	0.0196	0.0036	21113	5.48^**^
11	0.0092	0.0037	22557	2.47^*^
12	0.0276	0.0030	25518	9.20^**^
13	0.0072	0.0032	21703	2.26^*^
14	0.0216	0.0030	25657	7.12^**^
15	0.0236	0.0042	16572	5.61^**^
16	0.0188	0.0039	26990	4.78^**^

** p < 0.001,

* *p < 0.05. All models were estimated with an unstructured covariance matrix and the between-within method of estimating degrees of freedom. Significance levels are FDR corrected*.

**Figure 6:**
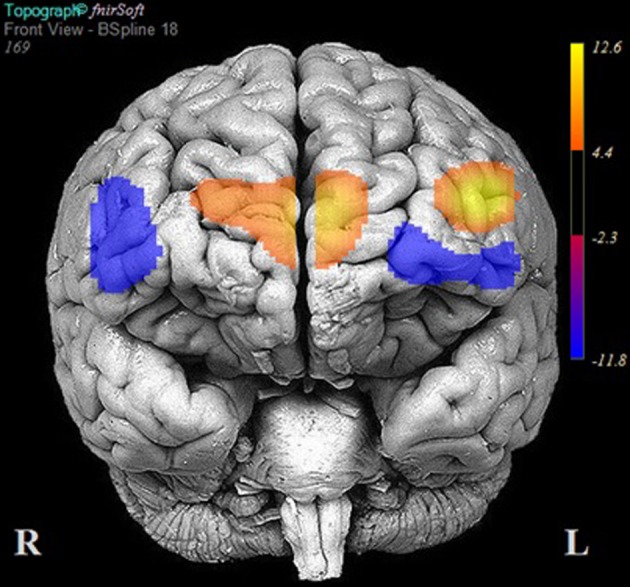
**Areas of significant activation and deactivation associated with solving two- and three-move problems on the Scarborough adaptation of the Tower of London (S-TOL) task for participants who did not meet the 90% accuracy threshold (*N* = 14)**. Areas of significant activation are denoted in red, and areas showing significant deactivation are in blue. Data represent *t*-scores for the contrast of multiple-move and zero-move conditions (*p* < 0.05, False-Discovery Rate-corrected).

Fourteen participants did not meet the 90% accuracy threshold. Table 6 reports the results of multilevel analyses comparing oxy-Hb across the MM and ZM conditions for these participants. Significant increases in oxy-Hb compared to ZM were observed in seven channels, encompassing the anterior aspects of the left middle/IFG (channels: 1 and 3), and the medial PFC (channels: 7, 8, 9, 10, and 11). Conversely, significant decreases in oxy-Hb compared to ZM were observed in six channels, encompassing the left middle/IFG (channels: 2, 4, and 6), and right middle/IFG (channels: 14, 15, and 16). Channels 5, 12, and 13 did not demonstrate a significant change in oxy-Hb across the MM and ZM conditions. These results are portrayed in Figure 6.

**Table 6:** **Multilevel analyses comparing oxy-Hb levels for participants who did not meet the 90% accuracy threshold (*N* = 14)**.

**fNIRS Channel**	***B***	***SE***	***df***	***t***
1	0.0805	0.0075	8880	10.67^**^
2	−0.0460	0.0083	10585	−5.48^**^
3	0.0649	0.0051	14635	12.64^**^
4	−0.0154	0.0065	12404	−2.36^*^
5	0.0006	0.0047	14207	0.14
6	−0.0359	0.0075	10494	−4.80^**^
7	0.0492	0.0052	13393	9.40^**^
8	0.0350	0.0068	12246	5.13^**^
9	0.0348	0.0056	13369	6.24^**^
10	0.0289	0.0064	12079	4.48^**^
11	0.0401	0.0068	8785	5.93^**^
12	−0.0055	0.0069	12392	−0.80
13	−0.0101	0.0055	12141	−1.85^*^
14	−0.0239	0.0062	13297	−3.82^**^
15	−0.0388	0.0068	8334	−5.66^**^
16	−0.0810	0.0068	14190	−11.84^**^

** p < 0.001,

* *p < 0.05. All models were estimated with an unstructured covariance matrix and the between-within method of estimating degrees of freedom. Significance levels are FDR corrected*.

### Ancillary analyses: deliberation and left DLPFC activation

Beyond analyses of accuracy on the S-TOL and its relationship to PFC activity on simple and complex problems, we examined the personality trait deliberation as an external validator of the aforementioned findings. As previously mentioned, deliberation refers to the tendency to think carefully before acting. The left DLPFC is crucial for problem-solving, specifically, the extraction of goal information and the generation of an internal problem representation. Accordingly, we predicted that deliberation would moderate the relationship between problem-solving and left DLPFC activation on the S-TOL. Specifically, we hypothesized that individuals higher in trait deliberation would show consistently high levels of left DLPFC activation across both higher and lower levels of task difficulty. Less deliberate individuals were hypothesized to demonstrate greater left DLPFC activation when solving problems of higher difficulty but less activation when solving simpler problems.

For this ancillary analysis, the left DLPFC was defined as our region of interest using channels 1, 2, 3, and 4. The time-marked data points from these channels were aggregated with list-wise deletion because some fNIRS data were missing at random. The aforementioned data screening and filtering yielded a total of 18, 305 data points across 21 participants for analysis of the left DLPFC. To test our hypothesis that Deliberation might moderate activity in the left DLPFC on the S-TOL, we estimated a multilevel model that examined the Level 1 effect of problem-solving, the Level 2 effect of Deliberation, and the problem-solving × Deliberation cross-level interaction in the prediction of oxy-Hb. The last term of this model was of particular interest because it tested whether or not within-person differences in oxy-Hb across the ZM and MM conditions varied as a function of between-person differences in trait Deliberation.

**Figure 7:**
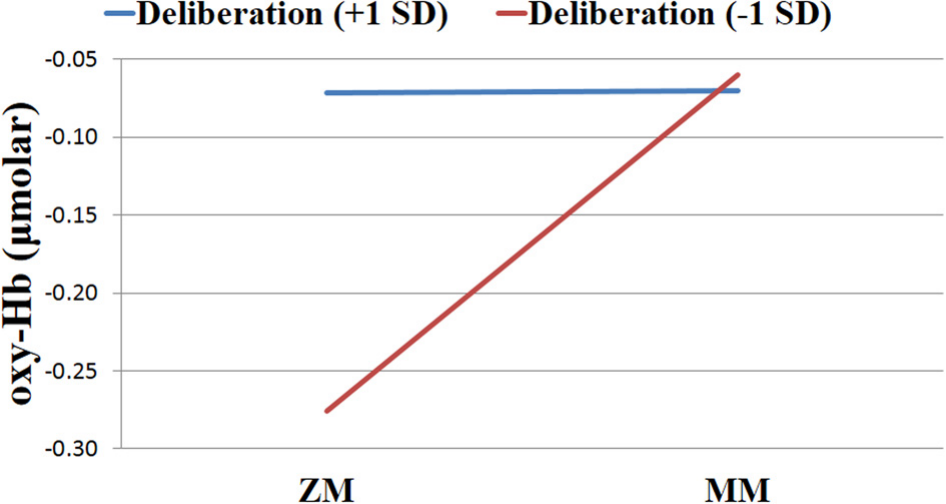
**Levels of oxygenated hemoglobin (oxy-Hb) for individuals high vs. low in Deliberation in the left dorsolateral prefrontal cortex across zero-move (ZM) and multiple-move (MM) conditions on the Scarborough adaptation of the Tower of London task**.

As expected, this analysis uncovered a significant cross-level interaction between problem-solving and Deliberation on the S-TOL [*b* = −0.01, *SE* = 0.00, *t*_(18282)_ = −12.51, *p* < 0.001]. In order to probe the nature of this significant interaction, the effect of problem-solving in the left DLPFC was examined at high (+1 *SD*) and low (−1 *SD*) levels of Deliberation (West and Aiken, 1991). As hypothesized, this analysis revealed that activation in the left DLPFC was higher in the MM relative to the ZM condition for participants who reported lower levels of Deliberation [*b* = 0.11, *SE* = 0.01, *t*_(18282)_ = 18.13, *p* < 0.0001], as compared to those who reported higher levels of this trait [*b* = 0.00, *SE* = 0.01, *t*_(18282)_ = 0.10, *p* = 0.92]. That is, whereas those participants who reported higher levels of Deliberation did not show a significant difference in oxy-Hb across the ZM and MM conditions, those participants who reported lower levels of this trait showed higher activation in the MM condition relative to the ZM condition. Furthermore, Deliberation was not significantly related to oxy-Hb during the MM condition [*b* = 0.00, *SE* = 0.01, *t*_(19)_, *p* = 0.92], but it was marginally associated with increased activation during the ZM condition [*b* = 0.01, *SE* = 0.01, *t*_(19)_ = 2.04, *p* = 0.06]. This latter result suggested that participants who self-reported higher levels of Deliberation may have been more engaged in problem-solving on the S-TOL even on ZM trials. This significant interaction is illustrated in Figure 7 and highlights that activation of the left DLPFC during the S-TOL varied predictably as a function of individual differences in Deliberation.

## Discussion

The present study described a new computerized version of the TOL (Shallice, 1982) designed to improve on the shortcomings of alternative variants of this task which were developed for neuroimaging purposes. Other computerized versions of this task employed in neuroimaging studies differed along several task parameters, including the number of moves required to achieve the target configuration, the mode of responding, and the baseline or “control” task used for comparison with more complex problem-solving trials. Variations in these important task attributes could lead to different findings across studies and obscure neural activation patterns associated with problem-solving on this task. The S-TOL was designed to include trials that required no more than three moves to achieve a goal configuration and responses were made using a simple two-alternative forced-choice modality. The baseline task utilized problems that required a minimum of zero moves to achieve the target configuration, and the same task instruction was employed on two- and three-move problems as for ZM problems in order to control for working memory load across task conditions. Using 16-channel fNIRS, activation of the PFC was evaluated by comparing hemodynamic changes in oxy-Hb for conditions requiring two or three moves with those requiring no moves to achieve the target configuration.

**Figure 8:**
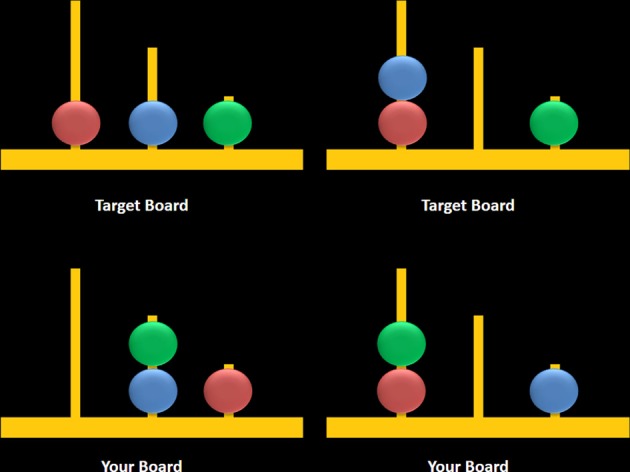
**Sample three-move problems from the Scarborough adaptation of the Tower of London task that are low (left) vs. high (right) in search depth**. These sample problems require a minimum of three moves to solve the problem and they either require an intermediate move (left) or do not require an intermediate move to achieve the target configuration (right).

Consistent with expectations, participants achieved high levels of accuracy on both two- and three-move problems on the S-TOL (~89%). When activation on these problems was contrasted with ZM problems, increases in oxy-Hb were observed primarily in the left DLPFC and right medial PFC. Conversely, decreased activation was observed in superior channels in the medial PFC and a single channel in the right IFG. The results of this study partly converge with prior neuroimaging research using other versions of the TOL which found largely bilateral DLPFC activation during the active completion of problems requiring two or more moves to achieve a goal configuration (Baker et al., 1996; Dagher et al., 1999; Newman et al., 2003; Boghi et al., 2006; Rasmussen et al., 2006; Wagner et al., 2006; Just et al., 2007; Den Braber et al., 2008; Fitzgerald et al., 2008; De Ruiter et al., 2009; Ruh et al., 2012). Bilateral engagement of the DLPFC during problem-solving on the TOL, however, has been challenged by research which suggests that the right and left homologs of this region may subserve distinct problem-solving functions, namely, those involved in search depth and goal hierarchy, respectively (Kaller et al., 2011). Search depth refers to the degree of interdependence between consecutive steps in problem-solving, whereas goal hierarchy reflects the degree to which the configuration of the goal state makes the order of single steps either clearly evident or ambiguous. The S-TOL was intentionally designed to contain problems that varied only in search depth (i.e., either no or one intermediate and interdependent move was required to achieve the goal configuration on three-move problems) (see Figure 8). All goal hierarchies, however, were unambiguous. Therefore, the observation of predominantly left DLPFC activation on the S-TOL is consistent with Kaller et al. (2011) which found similarly lateralized DLPFC activity on unambiguous problems with greater search depth. In addition, the present study extended these findings by revealing that increased activity in the right portion of the medial PFC may also play a role in solving unambiguous problems which vary in search depth.

Whereas increased activation was observed in primarily left DLPFC for two- and three-move problems, significant deactivation was also detected in dorsal aspects of the medial PFC and right IFG. Many studies using the TOL did not report on areas which showed significant deactivation on this task; however, Boghi et al. (2006) found pronounced reductions in activity in the medial PFC using a modified TOL task. Interestingly, comparisons of activation within the medial PFC and right IFG for highly accurate vs. less accurate participants revealed distinct patterns of activity in these regions: highly accurate individuals showed greater deactivation in the medial PFC, whereas less accurate participants showed less activation within the right IFG. It is important to note that these apparent differences in regional brain activity were observed even though the range of accuracy scores on the S-TOL was restricted.

An extensive body of research has shown that whereas the performance of attention-demanding cognitive tasks is typically associated with increased activation in the DLPFC and decreased activation in the medial PFC (Fox et al., 2005), states of passive rest and self-focused attention are typically associated with increased activity in the medial PFC (Gusnard et al., 2001). In light of these previous studies, we speculate that the observed differences in activation across levels of accuracy reflect differences in the degree to which participants cognitively immersed themselves in the S-TOL. Specifically, we suggest that participants who performed the S-TOL with greater accuracy may have subjectively engaged themselves in the S-TOL to a greater extent. Indeed, the medial PFC is a region that is crucial for self-referential thought (e.g., Abraham, 2013; Araujo et al., 2013; D'argembeau, 2013; Moran et al., 2013) and research from the field of motivational psychology highlights that transient decreases of self-focused attention are typically reported when people experience themselves as being subjectively immersed in an activity (Csikszentmihalyi, 1990). In keeping with these ideas about the presently observed differential pattern of PFC activity across levels of accuracy on the S-TOL, other fNIRS work has shown that more extensive deactivation in the medial PFC during active task performance is associated with greater success in inhibiting a motor response (Rodrigo et al., 2014). Moreover, using positron emission tomography, Beauchamp et al. (2003) asked participants to complete a computerized TOL over four separate scanning sessions to observe changes in neural systems associated with learning on this task. They found that as performance on the TOL improved over subsequent sessions, the medial PFC showed a concomitant decrease in activity. Future neuroimaging studies using the S-TOL (and other problem-solving tasks) should continue to examine how performance outcomes are associated with different patterns of neural activation across the PFC and should specifically consider the contribution of participants' degree of subjective task immersion and self-focused attention to medial PFC deactivation.

Reduced activity in the right IFG was an unexpected finding given that inhibition and attentional control, functions frequently ascribed to this region (Hampshire et al., 2010), are not typically referenced in theoretical models of problem-solving. Presumably, inhibitory control is an important component cognitive function in problem-solving ability—indeed, when solving a problem, individuals must evaluate and select one allowable operator from the larger set of allowable operators, which themselves must be inhibited at each step of the problem-solving process (Ward and Allport, 1997). To investigate the potential role of the right IFG in problem-solving, activation during MM vs. ZM problems on the S-TOL was compared between highly accurate and less accurate participants. Less accurate participants showed attenuated activity in the right IFG, whereas highly accurate participants displayed greater activation in this region. Accuracy on the S-TOL may therefore be subserved, at least in part, by the efficiency with which inhibitory processes are engaged during active problem-solving on items requiring at least two or three moves to achieve a target configuration. This speculation is also supported by neuropsychological research which indicates that accuracy on the TOL is strongly related to performance on tests of inhibitory control (Welsh et al., 1999; Miyake et al., 2000). It should be noted, however, that although lower levels activation were observed in the left and right IFG for less accurate participants, a region within the left IFG appeared to show higher activation. This perhaps suggests that the left IFG plays a unique role in problem-solving that extends beyond attention related task engagement. Future research should investigate this observation to further delineate the contributions of different regions of the PFC to problem-solving ability.

As an external validator of the current findings, patterns of left DLPFC activation on the S-TOL were examined in relation to a personality trait descriptor of decision-making, referred to as deliberation. This trait reflects the extent to which individuals think carefully before acting or speaking (McCrae and Costa, 2010). More deliberate individuals showed similar levels of activation in the left DLPFC on both lower and higher difficulty problems. These findings suggest that individuals who tend to think problems through carefully before acting may engage neural processes necessary for effective problem-solving regardless of task difficulty. Individuals that described themselves as less deliberate, however, had less activation in the left DLPFC while solving lower difficulty problems but higher activation on higher difficulty problems. Theoretically consistent associations between left DLPFC activation and individual differences in trait deliberation provide convergent validity for the S-TOL as a task that may effectively probe neural systems involved in problem-solving ability.

### Limitations and future directions

A number of limitations should be considered when interpreting the current findings. First, a greater number of females were recruited in this study. There is emerging evidence suggesting that females completing the TOL may show greater activation bilaterally in the DLPFC and right parietal cortex than their male counterparts (Boghi et al., 2006). Subsequent research should achieve a greater balance in female and male participants to evaluate possible differences between these groups in PFC activation. Second, the S-TOL did not include highly complex problems (i.e., those requiring a minimum of four or more moves to reach the goal configuration). This task was intentionally designed to evaluate problems requiring a minimum of two or three moves with the aim of ultimately translating the S-TOL to clinical populations that may have difficulty correctly answering more complex problems. Indeed, prior neuroimaging studies using variants of the TOL reported accuracy that ranged from 46 to 82% for five-move problems in non-clinical samples, although nearly one-third of studies published since 2000 did not report accuracy on the TOL (see Table 1). A difficulty with incorporating more complex problems in neuroimaging studies employing block designs is that lower levels of accuracy may be confounded with neural activation differences between clinical and non-clinical groups. The S-TOL achieved reasonably high levels of accuracy on two- and three-move problems, although generalizations derived from this task may be limited to relatively less complex problem-solving tasks. Third, larger sample sizes would provide more statistical power to evaluate the relationship between trait measures of problem-solving (e.g., deliberation) and neural activation on the S-TOL. The current exploratory findings provided preliminary support for the relationship of self-reported deliberation to left DLPFC activation on the S-TOL; however, examination of other potentially relevant personality traits (e.g., impulsiveness, excitement-seeking) would be permitted with suitably larger sample sizes. Indeed, fNIRS may hold potential to significantly advance personality neuroscience by providing researchers with a cost-effective tool to gather large sample sizes that are necessary to provide adequate power for personality-based research. Fourth, it should be noted that more research is needed to further validate the S-TOL above and beyond our initial evidence presented here, and to provide evidence demonstrating its specific advantages over other computerized TOL tasks. Furthermore, more research is needed with clinical samples to determine whether the S-TOL may indeed be suitable for translational research with these groups. Finally, the S-TOL and other conventional tower tasks are unable to identify the neural substrate underlying problems that are discovered or created. Rather, the S-TOL is a presented problem with clearly defined task parameters. It will be important for future neuroimaging studies to push the traditional boundaries of problem-solving research to explore the neural underpinnings involved in solving problems requiring discovery or creation by the problem-solver.

## Author contributions

Anthony C. Ruocco designed the study, developed the problem-solving task, and wrote the majority of the manuscript. Achala H. Rodrigo assisted in the development of the problem-solving task, conducted statistical analyses, and wrote portions of the manuscript. Jaeger Lam and Bryanna Graves wrote portions of the manuscript. Stefano I. Di Domenico and Hasan Ayaz assisted with statistical analysis and wrote portions of the manuscript.

### Conflict of interest statement

The optical brain imaging instrumentation utilized in the present research was manufactured by fNIR Devices, LLC. Dr. Hasan Ayaz was involved in the development of the technology and thus offered a minor share in fNIR Devices, LLC. All other authors declare that the research was conducted in the absence of any commercial or financial relationships that could be construed as a potential conflict of interest.

## Footnotes

^1^The authors of nearly all functional neuroimaging studies employing adapted versions of the Tower of London task described these tasks as measures of *planning*. Whereas paper-and-pencil tower tasks are often used to measure planning ability, the primary index of this cognitive function on these tasks is the duration of time that an examinee deliberates before physically attempting their first move on each problem. Computerized versions of these tasks adapted for functional neuroimaging studies typically did not ask examinees to physically manipulate the balls or discs. Instead, examinees were normally only required to indicate the minimum number of moves necessary to achieve each goal state. Therefore, these tasks typically did not directly measure planning ability and are referred to in this paper as tests of problem-solving ability.
